# Dynamic changes of soluble HLA-G and cytokine plasma levels in cervical cancer patients: potential role in cancer progression and immunotherapy

**DOI:** 10.1007/s00432-022-04331-4

**Published:** 2022-09-02

**Authors:** Hui-Hui Xu, You-You Xie, Jun-Gan, Zhi Yang, Qiu-Yue Han

**Affiliations:** 1 Medical Research Center, Taizhou Hospital of Zhejiang Province, Wenzhou Medical University, Linhai, Zhejiang People’s Republic of China; 2 Key Laboratory of Minimally Invasive Techniques and Rapid Rehabilitation of Digestive System Tumor of Zhejiang Province, Linhai, Zhejiang People’s Republic of China; 3 Radiotherapy Department, Taizhou Hospital of Zhejiang Province, Wenzhou Medical University, Linhai, Zhejiang People’s Republic of China; 4 Biological Resource Center, Taizhou Hospital of Zhejiang Province, Wenzhou Medical University, Linhai, Zhejiang People’s Republic of China

**Keywords:** Soluble HLA-G, Cytokine, Cervical cancer, Inflammation, Diagnosis, Immunotherapy

## Abstract

**Purpose:**

Chronic inflammation has been proven to be an important factor in carcinogenesis. Cytokines are the central mediators in the inflammatory microenvironment, and their release may be influenced by soluble HLA-G (sHLA-G). The aim of this study was to monitor the dynamic process of these soluble factors in patients with cervical cancer at Taizhou Hospital of Zhejiang Province, trying to understand their relationship with diagnosis, treatment, and prognosis.

**Methods:**

We quantified plasma levels of sHLA-G and 12 cytokines using ELISA and flow cytometry, respectively, in the peripheral blood of patients with cervical cancer divided into three groups: preoperation, postoperation and clinical relapse. Healthy women were used as the control group. Data were analysed by non-parametric tests, receiver-operating characteristic (ROC) curves, and Kaplan–Meier plotter (log-rank test).

**Results:**

In this study, our findings showed that preoperation plasma levels of sHLA-G and the cytokines IL-6, IL-10, and IFN-γ in cervical cancer patients had a good discriminatory effect between cervical cancer patients and healthy women. It should be noted that plasma levels of sHLA-G, IL-6, and IL-10 were significantly decreased within 30 days after radical hysterectomy (*P* < 0.05). A positive correlation was observed between IL-6 and IL-10, IL-8 and IL-17 levels preoperatively. In contrast, sHLA-G levels were negatively correlated with IL-10 but not with other cytokines. An increased survival rate in patients with cervical cancer was associated with IL-5 < 1.70 pg/mL, IL-17 < 2.30 pg/mL, and IFN-α < 2.26 pg/mL preoperatively. In addition, our findings showed that the levels of cytokines IL-6, IL-8, IL-12p70, IL-17, and IFN-γ may be related to 5-year relapse rates and/or the metastasis of cervical cancer.

**Conclusion:**

The current findings enhance our understanding of the dynamic process (preoperation, postoperation and clinical relapse) of sHLA-G and these cytokines in the plasma of patients with cervical cancer from diagnosis to prognosis. These biomarkers may play a potential therapeutic target role of such dynamic changes in the immunotherapy for cervical cancer.

**Supplementary Information:**

The online version contains supplementary material available at 10.1007/s00432-022-04331-4.

## Introduction

Chronic inflammation has been proven to be involved in several stages of carcinogenesis, including cellular transformation, proliferation, invasion, angiogenesis, and metastasis (Balkwill and Coussens 2004; Singh et al. 2019). Up to 20% of cancers are associated with chronic inflammation, such as the inflammatory microenvironment caused by human papillomavirus (HPV) infection in cervical epithelial cells, which may be the origin of cervical cancer (Hemmat and Bannazadeh Baghi 2019; Sadri Nahand et al. 2020). Cytokines are central mediators of inflammation and immunity, and their release may be influenced by soluble human leukocyte antigen-G (sHLA-G) (Viganò et al. 2003).

HLA-G is a novel immune checkpoint molecule that is ectopically expressed in tumour cells and has an antitumour immune function (Xu et al. 2020; Carosella et al. 2015). Unlike the other HLA class I antigens, the HLA-G primary transcript is alternatively spliced into seven mRNAs, which encode four membrane-bound (HLA-G1 ~ -G4) and three soluble (HLA-G5 ~ -G7) protein isoforms (Ishitani and Geraghty 1992). An increasing number of studies have shown that HLA-G expression is associated with disease progression in patients with cervical cancer (Xu et al. 2020; Li et al. 2012; Dong et al. 2010; Zheng et al. 2011). However, among these previous studies of cervical cancer, the expression of HLA-G was mainly evaluated in the cell membrane (mHLA-G) of malignant lesions, and much less attention was given to its secretion as soluble HLA-G (sHLA-G) into the bodily fluids. It is worth noting that sHLA-G levels are altered in various pathologies, including cancer, and are thus of high interest as a biomarker, especially for early cancer detection (Kessler et al. 2020). However, sHLA-G levels and its potential interaction with cytokine expression profiles have not yet been well explored in cervical cancer. In this study, we monitored the dynamic changes of sHLA-G and cytokines levels in the peripheral circulation of patients with cervical cancer, and explored their potential role in cancer progression and immunotherapy.

## Materials and methods

### Study population

A total of 187 cervical cancer patients were enrolled in this retrospective study, including 172 patients with initial diagnosis and 15 patients with clinical relapse within 5 years at Taizhou Hospital of Zhejiang Province from 2008 to 2019. Among patients with initial diagnosis, 129 blood samples were drawn on the day before the operation and 43 blood samples were drawn approximately 30 days after radical hysterectomy. Based on blood collection time, there were three groups in this study: preoperation, postoperation and clinical relapse. Plasma was isolated and stored at − 80 °C and used for sHLA-G and cytokine level evaluation. The study included patients with cervical cancer at initial diagnosis or those with clinical relapse within 5 years and excluded other cancers that invade the cervix, such as vaginal cancer and rectal cancer. The staging was performed according to the criteria of the International Federation of Gynaecology and Obstetrics (FIGO). In addition, 86 unrelated healthy women from our Health Management Center with no personal or family history of cancer were also enrolled in this study.

### sHLA-G ELISA

Plasma levels of sHLA-G (shedded HLA-G1 and HLA-G5) were quantified with sandwich ELISA kits (Cat# RD194070100R, sHLA-G kit; Exbio). Each sample (100 μL) was measured in triplicate, and total sHLA-G levels were determined using a Multiskan FC microplate reader (Thermo Scientific, Waltham, MA) at 450 nm. The final concentration was determined by optical density according to the six-point calibration curve (range: 3.91–125 Units/mL). When the concentration exceeded 125 U/mL, diluted samples were used, and the dilution factors were considered to calculate the sHLA-G concentration. The protocols were performed according to the manufacturer’s instructions.

### Cytokine measurement

A cytometric bead array was used to determine the presence of different cytokines in the plasma. A human cytokine assay kit was approved by China’s FDA (Certified No. 20180087), coated with 12 specific antibodies against 12 different cytokines, including IL-1β, IL-2, IL-4, IL-5, IL-6, IL-8, IL-10, IL-12p70, IL-17, interferon (IFN)-γ, IFN-α, and tumour necrosis factor (TNF)-α. Cytokine measurement was performed according to the manufacturer’s protocol. Briefly, 25 μl of tested plasma or diluent was incubated with 25 μl capture beads suspension and 25 μl antibodies at room temperature for 2 h, and then 25 μl phycoerythrin-conjugated streptavidin (SA-PE) reagent was added and incubated at room temperature for half an hour. After washing in 500 μl wash buffer, the samples were run using BD Canto II flow cytometer (BD Biosciences, San Jose, CA), and data were analysed using LEGENDplex 8.0 data analysis software. The limit of detection for each cytokine was 2.44–10,000 pg/mL. When the cytokine concentration was below the detection limit, it was considered 0 pg/mL.

### Statistical analysis

For statistical analysis, we used SPSS 16.0 (SPSS, Inc., Chicago, IL, USA) and GraphPad Prism 5.0 (GraphPad Inc., San Diego CA). *P* < 0.05 (two-tailed) was considered to be statistically significant. The relationship between categorical variables (including tumour subtypes, clinical staging, and tumour node metastasis) was assessed by the Chi-square (*χ*^2^) test. Numerical variables were analysed by the non-parametric Mann–Whitney *U* test. Differences in plasma levels of sHLA-G and cytokines were evaluated by the Kruskal–Wallis *H* test using data from patients with cervical cancer at initial diagnosis (preoperative plasma samples *n* = 129 or postoperative plasma samples *n* = 43) and at clinical relapse (plasma samples *n* = 15). Statistical associations between plasma levels of sHLA-G and cytokines were analysed by Spearman’s correlation coefficient.

The feasibility of using plasma sHLA-G as a potential biomarker for distinguishing patients with cervical cancer was assessed using receiver-operating characteristic (ROC) curve analysis. The areas under the ROC curve (AUCs) were calculated and subjected to statistical analysis. For the determination of the cutoff point in the plasma, the Youden index was adopted. Overall survival (OS) rates were evaluated from the date of diagnosis to the date of last follow-up (May 1, 2021) or date of patient death. Survival probabilities were calculated using Kaplan–Meier plotter. Differences between survival curves were analysed by the log-rank test.

## Results

### The characteristics of patients

The clinicopathological characteristics of cervical cancer patients in this study are shown in Table 1. A total of 187 patients were diagnosed with squamous cell carcinoma (including the keratinizing, non-keratinizing, and papillary subtypes), adenocarcinoma, and adenosquamous carcinoma. Among these patients, 172 (92.0%) patients were analysed at initial diagnosis, with a mean age of 57.7 years (range 33 ~ 91 years), of whom 50 (29.1%) had FIGO stage I, 80 (46.5%) had stage II, 41 (23.8%) had stage III and 1 (0.6%) had stage IV tumours. Among the patients analysed at initial diagnosis, 101 underwent surgery for lymph node dissection, and 34 (33.7%) had lymph node metastasis.

**Table 1:** The clinicopathological characteristics of patients with cervical cancer

Variables	Cervical cancer		Total (*n* = 187)
	Initial diagnosis (*n* = 172)	Clinical relapse (*n* = 15)	
Age (years)			
Mean ± SD	57.7 ± 13.1	58.8 ± 12.8	
Range	33 ~ 91	33 ~ 77	
Histological types			
Squamous cell carcinoma (SCC)	152 (88.37%)	13 (86.67%)	165 (88.24%)
Adenocarcinoma	16 (9.30%)	2 (13.33%)	18 (9.63%)
Adenosquamous carcinoma (ASC)	4 (2.33%)	0 (0.00%)	4 (2.14%)
FIGO Stage			
I	50 (29.07%)	1 (6.67%)	51 (27.27%)
II	80 (46.51%)	6 (40.00%)	86 (45.99%)
III	41 (23.84%)	8 (53.33%)	49 (26.20%)
IV	1 (0.58%)	0 (0.00%)	1 (0.53%)
Nodal status			
Negative	67 (38.95%)	2 (13.33%)	69 (36.90%)
Positive	34 (19.77%)	2 (13.33%)	36 (19.25%)
Unknown	71 (41.28%)	11 (73.33%)	82 (43.85%)
Follow-up			
Alive	139 (80.81%)	6 (40.00%)	145 (77.54%)
Death	33 (19.19%)	9 (60.00%)	42 (22.46%)

The follow-up period was 13 years or until death. The average follow-up time for all patients was 56 months (range 2 ~ 160 months), and during the entire period, there were 42 (22.5%) cancer-related deaths, including 6 (11.8%), 16 (18.6%) and 20 (40.8%) patients with FIGO stage I, stage II and stage III tumours, respectively.

### Patient sHLA-G and cytokine plasma levels

**Fig. 1:**
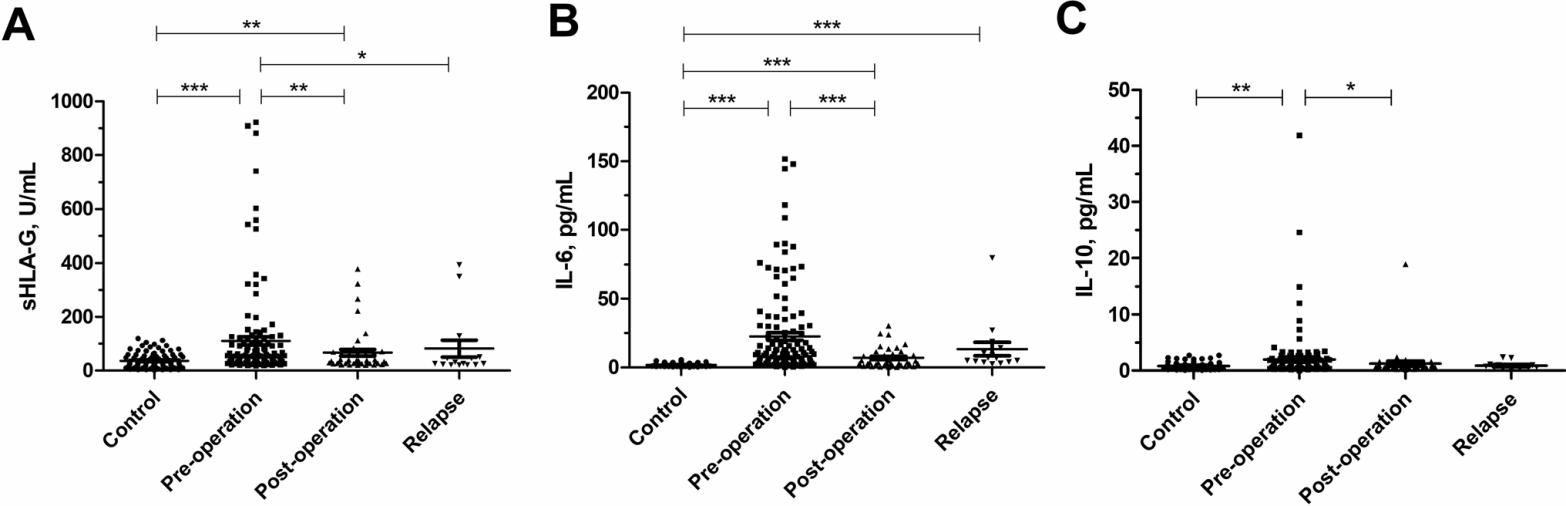
Changes in sHLA-G, IL-6, and IL-10 plasma levels before and after the initial surgery (preoperation, postoperation) and at clinical relapse. **A** sHLA-G, **B** IL-6, **C** IL-10. *P < 0.05, **P < 0.01, ***P < 0.001

Differences in plasma levels of sHLA-G and cytokines (median, 25th to 75th percentile) in cervical cancer patients are shown in Table 2. The Kruskal–Wallis *H* test was used to compare the dynamic changes of these soluble factors in the peripheral circulation of patients with cervical cancer, and the results revealed significant differences in sHLA-G (*P* = 0.004), IL-6 (*P* = 0.002), and IL-10 (*P* = 0.038) levels between the initial surgery (preoperation group, postoperation group) and clinical relapse group. As depicted in Fig. 1, plasma levels of sHLA-G, IL-6, and IL-10 were the highest at preoperation group and showed a significant decrease after radical hysterectomy (*P* < 0.05). However, there was no significant difference in plasma levels of sHLA-G between postoperation group and clinical relapse group. The plasma levels of other cytokines decreased slightly but not significantly after radical hysterectomy. In addition, there was no significant difference in plasma levels of sHLA-G between patients with squamous cell carcinoma and adenocarcinoma.

**Table 2:** Plasma levels of sHLA-G and cytokines in patients with cervical cancer at different disease stages

	Median of molecules values (25–75th)^a^			*P* value^b^
	Initial diagnosis		Clinical relapse (*n* = 15)	
	Blood collection before surgery (*n* = 129)	Blood collection after surgery (*n* = 43)		
IL-1β, pg/mL	UD (UD-2.42)^c^	UD (UD-2.42)	UD (UD-2.42)	0.870
IL-2, pg/mL	UD (UD-UD)	UD (UD-UD)	UD (UD-0.34)	0.116
IL-4, pg/mL	0.87 (0.78–1.00)	0.85 (0.80–0.96)	0.89 (0.80–1.00)	0.860
IL-5, pg/mL	1.70 (1.05–4.53)	1.36 (0.27–3.22)	1.36 (0.13–2.06)	0.074
IL-6, pg/mL	9.05 (3.79–26.48)	4.79 (2.35–8.68)	6.83 (4.21–12.42)	**0.002**
IL-8, pg/mL	3.88 (1.00–7.11)	2.75 (1.12–8.74)	5.63 (2.26–10.87)	0.242
IL-10, pg/mL	0.98 (0.57–1.74)	0.80 (0.45–1.03)	0.89 (0.53–0.98)	**0.038**
IL-12p70, pg/mL	1.30 (1.03–1.60)	1.24 (0.95–1.48)	1.19 (0.90–1.30)	0.158
IL-17, pg/mL	2.30 (1.40–3.14)	2.30 (1.08–4.07)	2.30 (1.40–3.97)	0.984
IFN-α, pg/mL	2.26 (1.86–2.89)	1.86 (1.49–2.89)	2.26 (1.77–2.46)	0.405
IFN-γ, pg/mL	4.18 (1.41–7.97)	4.18 (1.20–7.99)	3.61 (2.69–6.42)	0.977
TNF-α, pg/mL	0.57 (UD-1.63)	0.57 (UD-1.25)	0.57 (0.08–1.63)	0.790
sHLA-G, U/mL	50.86 (35.92–96.59)	34.77 (27.43–63.34)	26.76 (23.11–49.43)	**0.004**

*P *< 0.05 was indicated in bold

^a^Median, the 25% and the 75% percentile values

^b^*P* value calculated from Kruskal–Wallis *H* Test

^c^UD—undetectable levels

### Correlations of sHLA-G and cytokine levels prior to surgery

**Fig. 2:**
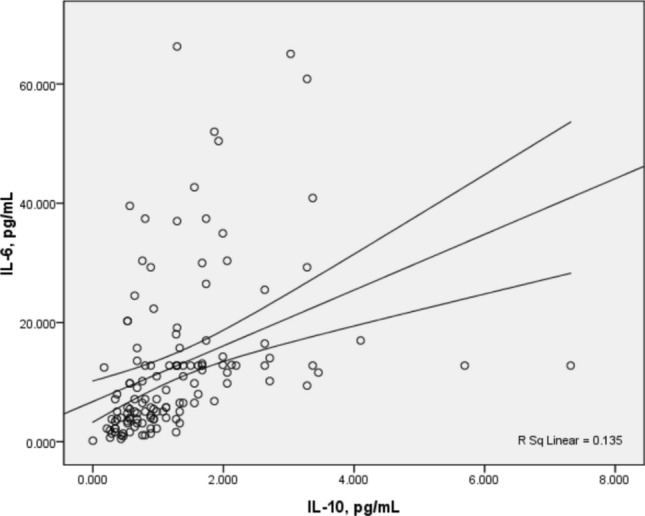
Correlation between preoperative IL-6 and IL-10 plasma levels

The systemic immune response of patients with cervical cancer indicated by these soluble factors and their potential roles in the clinical course of the disease is less clear. In this study, we further analysed the correlation between the expression levels of sHLA-G and the level of 12 cytokines in patients with cervical cancer. Spearman’s correlation analysis suggested significant positive associations between different cytokine plasma levels preoperatively (Table S1). It was observed that preoperative plasma IL-10 concentrations correlated directly with the levels of the multifunctional cytokine IL-6 (Spearman *r* 0.610, 95% CI 0.485–0.711) (Fig. 2), suggesting that IL-6 and IL-10 may play central mediator roles in cervical carcinogenesis. A positive correlation was observed between the plasma levels of IL-4, IL-5, IL-6, and IL-10, which may be indicative of the activation of the T helper (Th) 2 immune response (*P* < 0.05). Plasma levels of sHLA-G were negatively correlated with IL-10 preoperatively (*P* < 0.05) but not with other cytokines.

### Correlations of sHLA-G and cytokine levels after radical hysterectomy

After surgical removal of the cervical tumour, the dynamic balance of the cytokine network in the peripheral circulation of patients with cervical cancer was changed. Compared to preoperative observations, the correlations between the different cytokines changed significantly postoperatively (Table S2). Except for IL-17, the plasma levels of the other 11 cytokines and sHLA-G decreased either significantly or slightly after radical hysterectomy. A positive correlation was observed between IL-17 and IFN-γ levels, which suggested that there might be an inflammatory response in postoperative patients (*P* < 0.05). It was observed that postoperative plasma IL-4 concentrations correlated directly with IL-8 levels (Spearman *r* 0.600, 95% CI 0.357–0.767). Regardless of time (pre- or postoperative), a positive correlation was observed between plasma levels of IL-6 and IL-10 (*P* < 0.05), suggesting that these two cytokines may play a key role in immune regulation in cervical cancer.

In patients with relapsed cervical cancer, the correlations between different cytokines were not as obvious as those in patients in the initial diagnosis stage (Table S3). A positive correlation was observed between IL-6, IL-8, IL-12p70, IL-17, and IFN-γ levels (*P* < 0.05), suggesting that these cytokines may associated with 5-year relapse rates and/or the metastasis of cervical cancer. Interestingly, compared to preoperative findings, the correlation between IFN-α and IL-5 was reversed at relapse, changing from a positive correlation (Spearman *r* 0.317, *P* < 0.05) to a negative correlation (Spearman *r* − 0.665, *P* < 0.05).

### ROC analysis for sHLA-G and cytokines as biomarkers

**Fig. 3:**
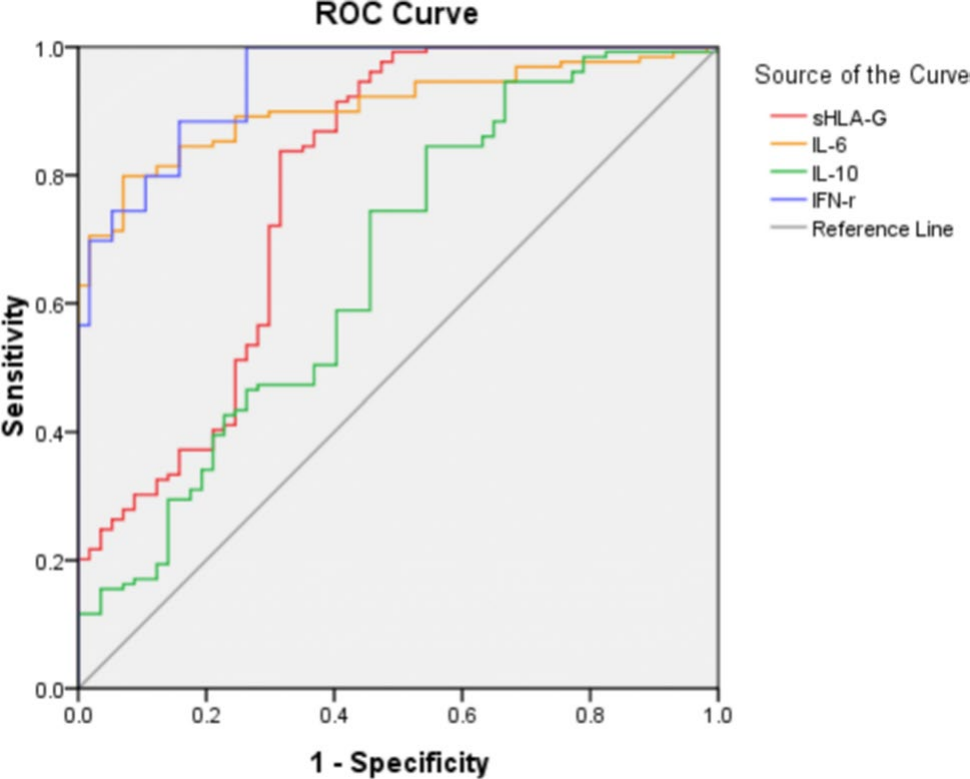
ROC analysis of sHLA-G and the cytokines IL-6, IL-10, and IFN-γ between cervical cancer patients and healthy women

To evaluate whether sHLA-G and cytokine plasma levels could discriminate cervical cancer patients from healthy women, we performed ROC analysis. As depicted in Fig. 3, ROC curves for sHLA-G and cytokines IL-6, IL-10, IFN-γ showed high areas under the curve, were 0.784 (95% CI 0.704–0.864), 0.906 (95% CI 0.865–0.948), 0.659 (95% CI 0.570–0.747), and 0.945 (95% CI 0.914–0.977), respectively. Notably, the plasma levels of sHLA-G, IL-6, and IL-10 were significantly decreased within 30 days after radical hysterectomy (*P* < 0.05) (Fig. 1). These findings showed that sHLA-G, IL-6, and IL-10 plasma levels had a good discriminatory effect between cervical cancer patients and healthy controls, suggesting that sHLA-G could be used as a potential biomarker of cervical cancer.

Preoperation plasma levels of sHLA-G were significantly higher in cervical cancer patients than that of healthy controls (110.86 ± 172.33 U/mL vs. 36.93 ± 29.30 U/mL, *P* < 0.05) (Fig. 1). The cutoff point of the highest Youden’s index was 52.14%. At the sHLA-G cutoff point of 33.75 U/mL, the pooled sensitivity and specificity were 83.72% and 31.58%, respectively.

### Effect of sHLA-G and cytokine levels on patient survival

To investigate the relationship between clinical parameters and the survival outcome of cervical cancer patients, patient survival outcomes relating to sHLA-G and cytokine plasma levels, patient age, surgery status, FIGO stage, and lymph node metastasis were further analysed (Table 3).

**Table 3:** Kaplan–Meier log-rank analysis of variables for cervical cancer patient overall survival outcomes

Variables	No. total	No. events	Survival (months) mean (95% CI)	*P* value
Age				
< 56 year	86	13	137.4 (126.2–148.7)	0.031
≥ 56 year	86	20	115.8 (97.9–133.8)	
FIGO				
Stage I	50	5	143.7 (130.2–157.1)	< 0.001
Stage II	80	13	130.8 (118.5–143.2)	
Stage III + IV	42	15	35.1 (29.8–40.4)	
Surgery status				
No surgery	63	18	37.6 (33.5–41.7)	< 0.001
Surgery	109	15	139.2 (129.5–148.9)	
Nodal status				
Negative	66	4	150.8 (143.0–158.6)	0.002
Positive	34	8	100.0 (82.2–117.6)	
Preoperation group (*n* = 129)				
IL-4				
Low level	75	12	134.8 (122.3–147.3)	0.053
High level	54	17	111.9 (93.3–130.6)	
IL-5				
Low level	67	11	134.6 (121.5–147.6)	0.064
High level	62	18	115.6 (98.3–132.9)	
IFN-α (FIGO stage I + II)				
Low level	54	10	130.2 (115.8–144.6)	0.439
High level	39	5	141.5 (126.4–156.6)	
IFN-α (FIGO stage III + IV)				
Low level	13	2	42.5 (35.6–49.5)	0.046
High level	23	12	28.7 (22.0–35.4)	
IL-17 (nodal status-negative)				
Low level	24	2	149.4 (136.7–162.2)	0.955
High level	22	2	150.2 (138.1–162.4)	
IL-17 (nodal status-positive)				
Low level	14	3	108.8 (87.0–130.5)	0.036
High level	4	3	49.8 (7.5–92.0)	
Postoperation group (*n* = 43)				
IL-1β				
Low level	24	1	82.0 (61.2–102.8)	0.038
High level	19	3	40.6 (35.0–46.2)	
IL-8				
Low level	23	1	82.0 (61.2–107.8)	0.062
High level	20	3	41.2 (36.0–46.3)	
IL-1β (FIGO stage I + II)				
Low level	22	1	82.0 (61.2–102.8)	0.067
High level	15	2	42.0 (36.9–47.1)	

In the initial diagnosis cohort (*n* = 172), clinical parameters such as patient age (*P* = 0.031), FIGO stage (*P* < 0.001), surgery status (*P* < 0.001) and nodal metastasis (*P* = 0.002) were significantly associated with overall survival (OS) outcomes in cervical cancer patients (Figure S1). As depicted in Figure S2, a shorter OS time for preoperative patients with cervical cancer was observed between IL-4^high^ and IL-4^low^ (111.9 months vs. 134.8 months; *P* = 0.053) and IL-5^high^ and IL-5^low^ (115.6 months vs. 134.6 months; *P* = 0.064). In addition, a shorter OS time for postoperative patients with cervical cancer was observed between IL-1β ^high^ and IL-1β ^low^ (40.6 months vs. 82.0 months; *P* = 0.038) and between IL-8^high^ and IL-8^low^ (41.2 months vs. 82.0 months; *P* = 0.062).

When cervical cancer patients were stratified by FIGO stages, IFN-α levels were only related to survival outcomes among cervical cancer patients with FIGO stages III + IV (28.7 months vs. 42.5 months; *P* = 0.046). In addition, IL-17 levels were the only factor related to survival outcomes among patients with positive nodal status (49.8 months vs. 108.8 months; *P* = 0.036) (Figure S2).

## Discussion

Cervical cancer, a common type of female cancer that arises at the opening of the uterus, is believed to be accelerated by inflammation. As HPV infection is the main cause of this cancer, this virus might initiate inflammation and expedite the process of cervical cancer (Hemmat and Bannazadeh Baghi 2019; Sadri Nahand et al. 2020). In addition to the local inflammation induced by HPV, the production of various cytokines and chemokines from the host innate and adaptive immune systems are also involved in the cervical carcinogenesis (Carrero et al. 2021; Paradkar et al. 2014; Alves et al. 2018). Systemic inflammation enhances the mobilisation of additional cells from the central and peripheral immune systems to participate in HPV clearance/persistence and/or immune suppressive responses (Rossi et al. 2021). Cellular immunity plays a key role in the defence mechanism of viral infections. Starting from naïve T cells, CD4^+^ T cells can differentiate into various effector cell subsets with specialised functions, including Th1, Th2, Th17, regulatory T (Treg) and T follicular helper (Tfh) cells (Basu et al. 2021). Th1 cytokines (IL-2, IL-12, IFN-γ, TNF-α, etc.) mainly induce cell-mediated immunity and have a beneficial effect in reducing cervical lesions, while Th2 cytokines (IL-4, IL-5, IL-6, IL-8, IL-10, etc.) and Th17 cytokines (IL-17, IL-22, etc.) have the potential to create a favourable environment for tumour development (Carrero et al. 2021). In addition, immune checkpoint HLA-G expression was negative in normal or adjacent non-tumorous tissues but was significantly increased along with CIN grade and cervical cancer metastasis, especially in HPV-infected tissues (Li et al. 2012; Dong et al. 2010). HLA-G expression and inhibitory cytokines’ secretion may promote local immunosuppression tumour microenvironment, which favours HPV persistence and tumour transformation (Xu et al. 2020; Aggarwal et al. 2020). Recently, the first anti-HLA-G chimeric antigen receptor (CAR)-T cells targeting HLA-G, which is both a tumour-specific antigen and an immune checkpoint molecule, could specifically target and eliminate both tumour cells and HLA-G^+^-suppressive cells (Anna et al. 2021). However, the development of CAR technology in gynaecologic malignancies is still in its early stage. To date, the results of three studies focussing on CAR-T cells therapy of cervical cancer are disappointing (Schepisi et al. 2021). In addition, soluble HLA-G released by tumour cells is unlikely to act as a decoy and significantly block anti-HLA-G CAR-T cells’ functions (Anna et al. 2021). Therefore, it is time to monitor the dynamic process of sHLA-G and cytokines in patients with cervical cancer, especially in the context of an excessive inflammatory response, such as cytokines storm observed after CAR-T cells’ therapy.

In this study, we reported that soluble HLA-G and specific cytokines levels were associated with the carcinogenesis and progression of cervical cancer from diagnostics to prognosis, including the following findings: (i) sHLA-G and cytokines IL-6, IL-10 plasma levels had a good discriminatory effect between cervical cancer patients and healthy women, suggesting that sHLA-G could be used as a potential biomarker of cervical cancer; (ii) after surgical removal of the cervical tumour, the correlations between the different cytokines changed significantly. The levels of IL-6 and IL-10 were positively correlated at the pre- and postoperation stages, suggesting that these two cytokines may play a key role in immune regulation in cervical cancer; (iii) at the relapse phase, the levels of IL-6, IL-8, IL-12p70, IL-17, and IFN-γ were the positively correlated, suggesting that these cytokines may associated with 5-year relapse rates and/or the metastasis of cervical cancer; and (iv) in the initial diagnosis cohort, high levels of IL-17 were associated with shorter overall survival times among patients with positive nodal status. These findings enhance our understanding of the dynamic process (preoperation, postoperation and clinical relapse) of sHLA-G and these cytokines in the plasma of patients with cervical cancer from diagnosis to prognosis. These biomarkers may play a potential therapeutic target role of such dynamic changes in the immunotherapy for cervical cancer.

HLA-G molecules has comprehensive immunosuppressive properties that are exerted in multiple steps to weaken the antitumour immune responses by acting on immune cells (T cells, NK cells, monocytes, dendritic cells, etc.) through its inhibitory receptors (ILT2, ILT4, KIR2DL4, etc.) (Xu et al. 2020; Carosella et al. 2015). The HLA-G/ILTs axis has been recently recognised as a novel immune checkpoint which offer a promising perspective for advance solid cancer immunotherapy (Anna et al. 2021; Lin and Yan 2021; Krijgsman et al. 2020; Schwich et al. 2020). Our previous study has been showed HLA-G expression increased progressively from pre-malignant to malignant cervical lesions, and HLA-G expression in FIGO stage I, stage II, and stage III + IV was 53.6%, 76.3% and 100.0%, respectively (*P* < 0.05) (Li et al. 2012). However, in the present study, there was no significant difference between FIGO stages (*P* = 0.51), tumour metastasis rates (*P* = 0.27) or overall survival rates (*P* = 0.81) and circulating sHLA-G level in patients with cervical cancer, regardless of histology. It is known that sHLA-G molecule influences directly and/or indirectly the growth of malignant tumours (Carosella et al. 2015; Kessler et al. 2020). We speculate that these differences may be caused by the functional differences between membrane-bound HLA-G and soluble HLA-G isoforms, or caused by the specific tumour microenvironment. Moreover, our findings consistent with previous studies, plasma levels of sHLA-G were commonly significantly higher in cervical cancer patients than in healthy women (Xu et al. 2020; Zheng et al. 2011; Aggarwal et al. 2020). It is worth to noting that plasma levels of sHLA-G decreased significantly within 30 days after radical hysterectomy, as well as IL-6 and IL-10. However, there was no significant difference in plasma levels of sHLA-G between postoperation and clinical relapse. Therefore, sHLA-G could not only be used as a novel tumour biomarker for the early diagnosis of patients at high risk of developing cervical cancer, but also seems to play an important role in monitoring the postoperative effects after radical hysterectomy.

Currently, little is known about the dynamic process of circulating sHLA-G and cytokines levels in patients with cervical cancer since HPV infection. A better insight into the relationship between checkpoint HLA-G and cytokines is to more effectively implement these tumour immunotherapy approaches, including anti-HLA-G neutralising antibodies and anti-HLA-G CAR-T cells (Carosella et al. 2021). It is well known that persistent infection by high-risk HPV is the main cause of cervical cancer and its precursor lesions (Hemmat and Bannazadeh Baghi 2019). Circulating IL-6 and IL-10 levels were increased in HPV-positive patients without cervical lesions, but IL-6 levels were higher in exfoliated cervical cells in the same patients, suggesting that IL-6 is mainly produced at the local site of HPV infection during the early inflammatory response and that the immune response changes at the systemic level (Bonin-Jacob et al. 2021). The median IL-6 level in HPV-positive patients was 3.40 (2.40–4.40) pg/mL in Bonin-Jacob et al. study (Bonin-Jacob et al. 2021), and in our study that in cervical cancer patients was 9.05 (3.79–26.48) pg/mL, it decreased significantly to 4.79 (2.35–8.68) pg/mL after radical hysterectomy and increased slightly to 6.83 (4.21–12.42) pg/mL at the relapse phase. However, we did not find any correlation between IL-6 levels and overall survival outcomes in cervical cancer patients. These findings suggested that IL-6 may play a key role in establishing HPV persistence, promoting tumour growth and metastasis (Carrero et al. 2021).

Moreover, the expression regulation mechanism between sHLA-G and IL-6 and IL-10 has been confirmed in vitro and in vivo (Liang et al. 2008; Gregori et al. 2010). Soluble HLA-G modulates ILT4-positive DCs’ results in recruitment of SHP-1 and SHP-2, increasing IL-6 production and conferring DCs with tolerogenic properties via the IL-6-STAT3 pathway (Liang et al. 2008). DC-10s is a subset of tolerogenic DCs which express high levels of HLA-G and secrete IL-10 and IL-6 in peripheral blood of patients with gastric cancer (Xu et al. 2016). DC-10s can induce T regulatory type 1 (Tr1) cells differentiation through the IL-10-dependent HLA-G/ILT4 pathway (Gregori et al. 2010). sHLA-G can induce the production of CD4+ inhibitory T cells through Fas/FasL pathway (Naji et al. 2007). These findings suggested that circulating sHLA-G may contribute to systemic immunosuppression, so that malignant cells escape immune killing.

At diagnosis, our findings showed that plasma levels of sHLA-G and IL-5, IL-6, IL-8, IL-10, IL-17, and were significantly increased in patients with cervical cancer. Based on these results, the following models for immune escape of cervical cancer cells can be hypothesised: following the HPV infection, sHLA-G expression may be induced and shift the cytokines expression profile towards Th2 in the peripheral blood circulation (Viganò et al. 2003; Xu et al. 2020; Almeida et al. 2018; Carosella et al. 2011); this in turn may further promote immunosuppression by upregulating HLA-G expression, including DC-10s, Treg, and MDSCs (Liang et al. 2008; Gregori et al. 2010; Xu et al. 2016; Naji et al. 2007; Urosevic and Dummer 2003; Pistoia et al. 2007). In addition, IL-6 is mainly produced at the local site of the cervix during the early inflammatory response, and the immune response gradually changes at the overall systemic level during cervical carcinogenesis (Hirano 2021). Therefore, it would be worthwhile to monitor the dynamic process of sHLA-G and cytokines in patients with cervical cancer and explore anti-HLA-G-based immunotherapy strategies for breaking down tolerance in cancer (Paradkar et al. 2014; Gimenes et al. 2014).

After radical hysterectomy, the dynamic balance of the cytokine network in the peripheral circulation of patients with cervical cancer was significantly changed. In particular, the plasma levels of sHLA-G, IL-6, and IL-10 were significantly decreased within 30 days after surgery, which provided strong evidence of the biological function of sHLA-G, IL-6, and IL-10 in cervical carcinogenesis. At the relapse phase, the plasma levels of IL-8 increased to 5.63 (2.26–10.87) pg/mL compared with 2.75 (1.12–8.74) pg/mL in the postoperative group, suggesting IL-8 may associated with tumour growth and metastasis. To the best of our knowledge, our result is one of the few reports of the negative correlation between HLA-G and IL-10. This discrepancy may be explained by the difference in cytokine-regulating functions between membrane-bound HLA-G and soluble HLA-G isoforms. In addition, the molecular mechanisms underlying the differential expression of HLA-G isoforms may be influenced by the tumour microenvironment, such as the surrounding cytokine profile (Almeida et al. 2018; Carosella et al. 2011; Urosevic and Dummer 2003). Our results showed that there was a negative correlation between sHLA-G and IL-10 plasma levels among these patients. Similar to our results, Almeida et al. revealed an inverse relationship between the levels of sHLA-G and IL-10 in paediatric patients with T cell acute lymphoblastic leukaemia (T-ALL) (Almeida et al. 2018). In the future, there will be a need for additional studies to obtain deeper insight into the association between HLA-G isoforms and cytokines expression profile in tumour microenvironment.

The limitations of this study are as follows: (i) lack of specific functional assessments focussed on an exploration of sHLA-G/cytokines and their receptors; such analysis might help to clarify and corroborate our findings; and (ii) lack of identification of immune cell types secreting specific cytokines; assessing these cell types might help for the development of novel immunological therapeutic approaches for cervical cancer.

In conclusion, the current findings enhance our understanding of the dynamic process of sHLA-G and cytokines in cervical carcinogenesis. sHLA-G could not only be used as a novel tumour biomarker for the early diagnosis of patients at high risk of developing cervical cancer, but also seems to play an important role in monitoring the postoperative effects after radical hysterectomy. Moreover, further illumination of the relationship between different HLA-G isoforms and cytokine profiles is meaningful and could be used to better select therapeutic targets to restore the patient’s antitumour response.

## Supplementary Information

Below is the link to the electronic supplementary material.

Supplementary file1 (TIF 13835 KB)

Supplementary file2 (TIF 13835 KB)

Supplementary file3 (DOC 20 KB)

Supplementary file4 (DOC 20 KB)

Supplementary file5 (DOC 19 KB)

## Data Availability

The datasets generated and analysed during the current study are available from the corresponding author, HHX, upon reasonable request.

## Abbreviations

CAR-T: Chimeric antigen receptor-T
FIGO: International Federation of Gynaecology and Obstetrics
HPV: Human papillomavirus
IFN-γ: Interferon-γ
OS: Overall survival
ROC: Receiver-operating characteristic
sHLA-G: Soluble human leukocyte antigen-G
TNF-α: Tumour necrosis factor-α
Treg: Regulatory T
