# Supplementary figures and images for: Dynamic changes of soluble HLA-G and cytokine plasma levels in cervical cancer patients: potential role in cancer progression and immunotherapy
Source: J Cancer Res Clin Oncol. 2022 Sep 2;149(8):4195–204. doi: 10.1007/s00432-022-04331-4 (PMC10349748; doi:10.1007/s00432-022-04331-4)

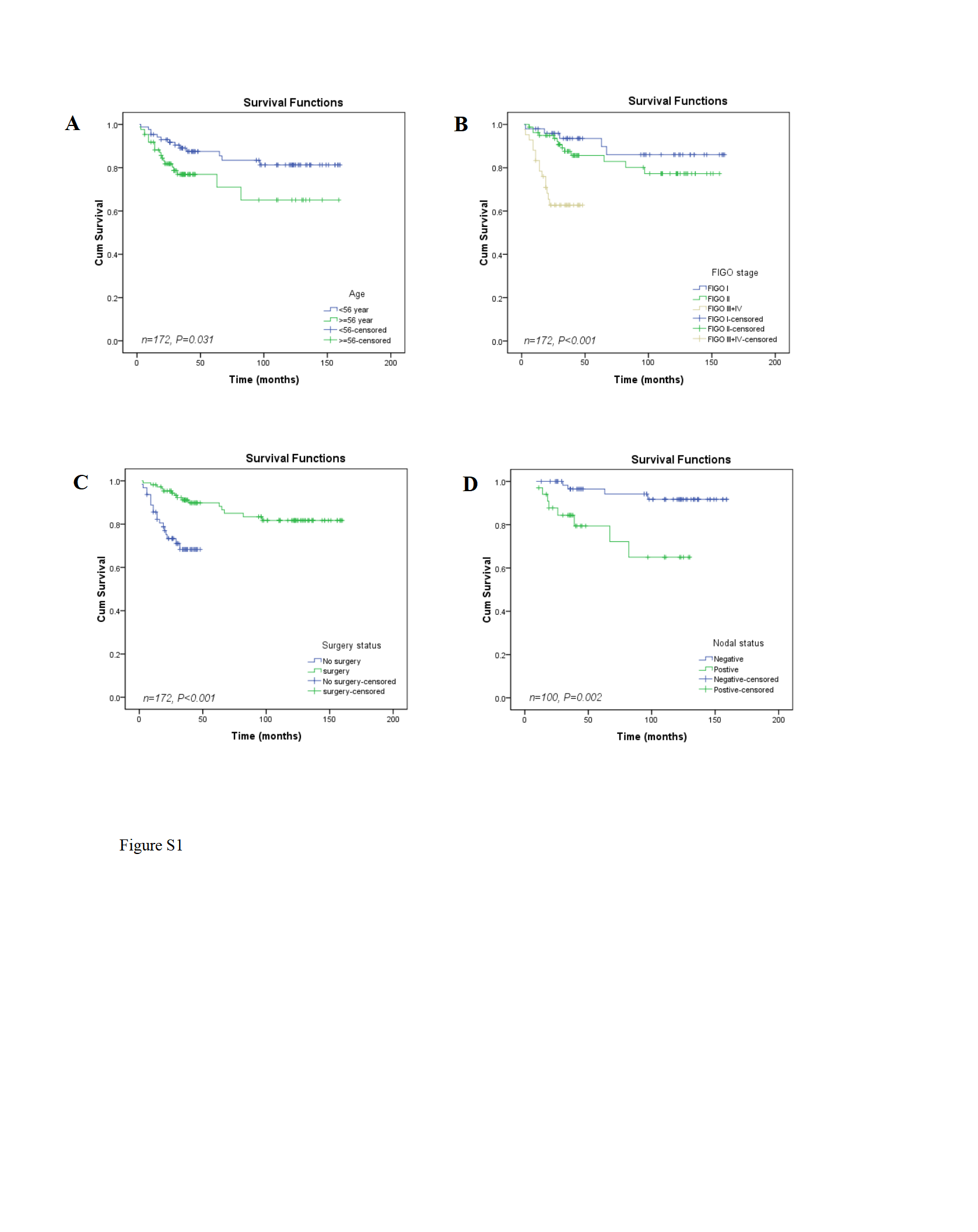

Supplement: Supplementary file 1 — Supplementary file1 (TIF 13835 KB) [file 432_2022_4331_MOESM1_ESM.tif]

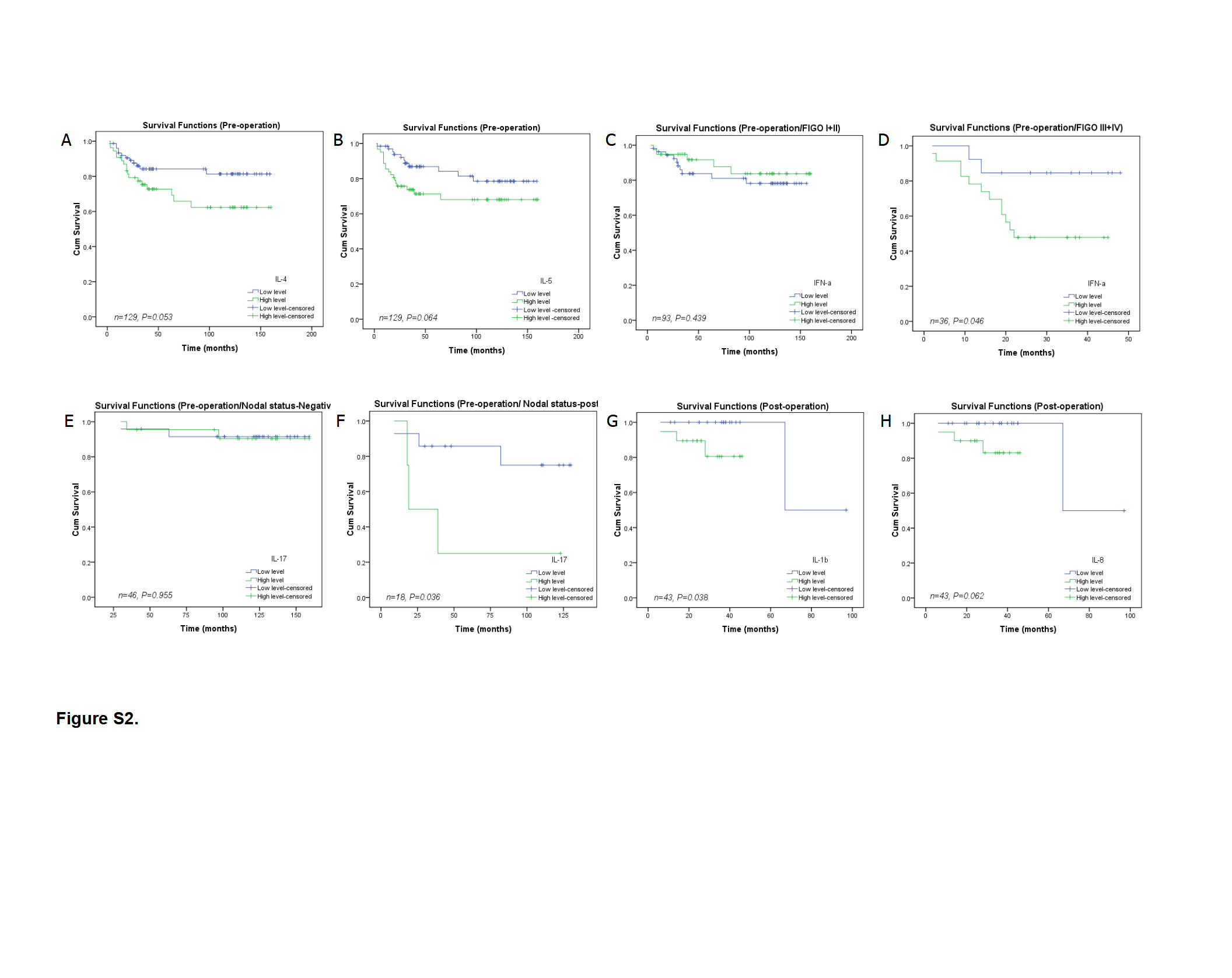

Supplement: Supplementary file 2 — Supplementary file2 (TIF 13835 KB) [file 432_2022_4331_MOESM2_ESM.tif]
