# Supplementary material for: Dynamic changes of soluble HLA-G and cytokine plasma levels in cervical cancer patients: potential role in cancer progression and immunotherapy
Source: J Cancer Res Clin Oncol. 2022 Sep 2;149(8):4195–204. doi: 10.1007/s00432-022-04331-4 (PMC10349748; doi:10.1007/s00432-022-04331-4)
Supplement: Supplementary file 3 — Supplementary file3 (DOC 20 KB) [file 432_2022_4331_MOESM3_ESM.doc]

| Table S1. Correlations between sHLA-G and different cytokines levels at diagnosis (preoperation,, n=129) | | | | | | | | | | | | | | |
| --- | --- | --- | --- | --- | --- | --- | --- | --- | --- | --- | --- | --- | --- | --- |
|  |  | IL-1β | IL-2 | IL-4 | IL-5 | IL-6 | IL-8 | IL-10 | IL-12 | IL-17 | IFN-α | IFN-γ | TNF-α | sHLA-G |
| Spearman's rho | IL-1β | 1.000 |  |  |  |  |  |  |  |  |  |  |  |  |
|  | IL-2 | .317^**^ | 1.000 |  |  |  |  |  |  |  |  |  |  |  |
|  | IL-4 | .164 | .187^*^ | 1.000 |  |  |  |  |  |  |  |  |  |  |
|  | IL-5 | .242^**^ | .286^**^ | .161 | 1.000 |  |  |  |  |  |  |  |  |  |
|  | IL-6 | -.160 | .010 | .016 | .141 | 1.000 |  |  |  |  |  |  |  |  |
|  | IL-8 | .284^**^ | .171 | .231^**^ | .109 | .021 | 1.000 |  |  |  |  |  |  |  |
|  | IL-10 | .086 | .218^*^ | .055 | .280^**^ | .610^**^ | .173^*^ | 1.000 |  |  |  |  |  |  |
|  | IL-12 | .240^**^ | .128 | .360^**^ | .174^*^ | .018 | .295^**^ | .201^*^ | 1.000 |  |  |  |  |  |
|  | IL-17 | .222^*^ | .204^*^ | .340^**^ | .211^*^ | .142 | .362^**^ | .148 | .237^**^ | 1.000 |  |  |  |  |
|  | IFN-α | .238^**^ | .206^*^ | .274^**^ | .317^**^ | .158 | .249^**^ | .291^**^ | .197^*^ | .251^**^ | 1.000 |  |  |  |
|  | IFN-γ | .125 | .047 | .410^**^ | .244^**^ | .045 | .250^**^ | .030 | .296^**^ | .337^**^ | .198^*^ | 1.000 |  |  |
|  | TNF-α | .196^*^ | .157 | .235^**^ | .191^*^ | -.114 | .252^**^ | .114 | .214^*^ | .309^**^ | .341^**^ | .307^**^ | 1.000 |  |
|  | sHLA-G | -.118 | .073 | -.089 | .053 | -.155 | -.173 | -.183^*^ | -.141 | -.102 | .025 | -.015 | .073 | 1.000 |
| *. Correlation is significant at the 0.05 level (2-tailed). | | | | | | |  |  |  |  |  |  |  |  |
| **. Correlation is significant at the 0.01 level (2-tailed). | | | | | | |  |  |  |  |  |  |  |  |
