# Supplementary material for: Dynamic changes of soluble HLA-G and cytokine plasma levels in cervical cancer patients: potential role in cancer progression and immunotherapy
Source: J Cancer Res Clin Oncol. 2022 Sep 2;149(8):4195–204. doi: 10.1007/s00432-022-04331-4 (PMC10349748; doi:10.1007/s00432-022-04331-4)
Supplement: Supplementary file 4 — Supplementary file4 (DOC 20 KB) [file 432_2022_4331_MOESM4_ESM.doc]

| Table S2. Correlations between sHLA-G and different cytokines levels at diagnosis (postoperation, n=43) | | | | | | | | | | | | | | | | | | | | | | |
| --- | --- | --- | --- | --- | --- | --- | --- | --- | --- | --- | --- | --- | --- | --- | --- | --- | --- | --- | --- | --- | --- | --- |
|  |  | IL-1β | IL-2 | IL-4 | IL-5 | IL-6 | IL-8 | | IL-10 | | IL-12 | | IL-17 | | IFN-α | | IFN-γ | | TNF-α | | sHLA-G | |
| Spearman's rho | IL-1β | 1.000 |  |  |  |  |  | |  | |  | |  | |  | |  | |  | |  | |
|  | IL-2 | .008 | 1.000 |  |  |  |  | |  | |  | |  | |  | |  | |  | |  | |
|  | IL-4 | .176 | .280 | 1.000 |  |  |  | |  | |  | |  | |  | |  | |  | |  | |
|  | IL-5 | .318^*^ | -.239 | -.100 | 1.000 |  |  | |  | |  | |  | |  | |  | |  | |  | |
|  | IL-6 | .310^*^ | .122 | .263 | .267 | 1.000 |  | |  | |  | |  | |  | |  | |  | |  | |
|  | IL-8 | .356^*^ | .257 | .600^**^ | -.163 | .288 | 1.000 | |  | |  | |  | |  | |  | |  | |  | |
|  | IL-10 | .326^*^ | .177 | -.010 | .341^*^ | .475^**^ | .154 | | 1.000 | |  | |  | |  | |  | |  | |  | |
|  | IL-12 | .116 | .100 | -.078 | .329^*^ | .244 | .028 | | .359^*^ | | 1.000 | |  | |  | |  | |  | |  | |
|  | IL-17 | .267 | .052 | .278 | .255 | .344^*^ | .398^**^ | | .363^*^ | | .266 | | 1.000 | |  | |  | |  | |  | |
|  | IFN-α | .055 | .379^*^ | .112 | -.008 | .089 | .177 | | .197 | | .486^**^ | | .296 | | 1.000 | |  | |  | |  | |
|  | IFN-γ | .346^*^ | -.086 | .109 | .153 | .264 | .269 | | .311^*^ | | .220 | | .454^**^ | | .121 | | 1.000 | |  | |  | |
|  | TNF-α | .016 | .304^*^ | .084 | -.025 | .074 | .362^*^ | | .075 | | .133 | | .200 | | .176 | | .241 | | 1.000 | |  | |
|  | sHLA-G | .003 | .080 | -.144 | .139 | -.042 | -.295 | | -.069 | | .103 | | -.251 | | .020 | | -.211 | | .077 | | 1.000 | |
| *. Correlation is significant at the 0.05 level (2-tailed). | | | | | | |  |  | |  | |  | |  | |  | |  | |  | |  |
| **. Correlation is significant at the 0.01 level (2-tailed). | | | | | | |  |  | |  | |  | |  | |  | |  | |  | |  |
