# Supplementary material for: Dynamic changes of soluble HLA-G and cytokine plasma levels in cervical cancer patients: potential role in cancer progression and immunotherapy
Source: J Cancer Res Clin Oncol. 2022 Sep 2;149(8):4195–204. doi: 10.1007/s00432-022-04331-4 (PMC10349748; doi:10.1007/s00432-022-04331-4)
Supplement: Supplementary file 5 — Supplementary file5 (DOC 19 KB) [file 432_2022_4331_MOESM5_ESM.doc]

| Table S3. Correlations between sHLA-G and different cytokines levels at clinical relapse ( n=15) | | | | | | | | | | | | | | | | |
| --- | --- | --- | --- | --- | --- | --- | --- | --- | --- | --- | --- | --- | --- | --- | --- | --- |
|  |  | IL-1β | IL-2 | IL-4 | IL-5 | IL-6 | IL-8 | IL-10 | IL-12 | IL-17 | | IFN-α | | IFN-γ | TNF-α | sHLA-G |
| Spearman's rho | IL-1β | 1.000 |  |  |  |  |  |  |  |  | |  | |  |  |  |
|  | IL-2 | .357 | 1.000 |  |  |  |  |  |  |  | |  | |  |  |  |
|  | IL-4 | .155 | .008 | 1.000 |  |  |  |  |  |  | |  | |  |  |  |
|  | IL-5 | .163 | .303 | .188 | 1.000 |  |  |  |  |  | |  | |  |  |  |
|  | IL-6 | .248 | .322 | .056 | .024 | 1.000 |  |  |  |  | |  | |  |  |  |
|  | IL-8 | .435 | .379 | .078 | .076 | .360 | 1.000 |  |  |  | |  | |  |  |  |
|  | IL-10 | .102 | -.059 | -.169 | -.113 | .096 | .451 | 1.000 |  |  | |  | |  |  |  |
|  | IL-12 | .467 | .449 | .296 | .134 | .238 | .672^**^ | .250 | 1.000 |  | |  | |  |  |  |
|  | IL-17 | .478 | .346 | .225 | .206 | .725^**^ | .577^*^ | .453 | .335 | 1.000 | |  | |  |  |  |
|  | IFN-α | -.122 | -.171 | -.157 | -.665^**^ | -.025 | .168 | -.197 | .085 | -.153 | | 1.000 | |  |  |  |
|  | IFN-γ | .155 | .383 | .261 | -.114 | .451 | .226 | .238 | .564^*^ | .458 | | .072 | | 1.000 |  |  |
|  | TNF-α | .015 | .091 | .078 | .298 | -.128 | .044 | .149 | .339 | -.270 | | -.326 | | .123 | 1.000 |  |
|  | sHLA-G | .138 | -.091 | .444 | .486 | .072 | -.075 | -.259 | .207 | .005 | | -.221 | | -.009 | .318 | 1.000 |
| *. Correlation is significant at the 0.05 level (2-tailed). | | | | | | |  |  |  |  | |  | |  |  |  |
| **. Correlation is significant at the 0.01 level (2-tailed). | | | | | | |  |  |  |  |  | |  | |  |  |
